# Protocol to develop a specialised curriculum in primary care cancer research in an Irish medical school

**DOI:** 10.12688/hrbopenres.13911.1

**Published:** 2024-10-08

**Authors:** Logan Verlaque, Benjamin Jacob, Kurdo Araz, Aileen Barrett, Fiona Kent, Patrick Redmond

**Affiliations:** 1 Department of General Practice, Royal College of Surgeons in Ireland, Dublin, Ireland; 2 Irish College of General Practitioners, Dublin, Ireland; 3 Health Professions Education Centre, Royal College of Surgeons in Ireland, Dublin, Ireland

**Keywords:** Curriculum; Education, Medical; Program Development; Needs Assessment; Research Design; Primary Care; Cancer Research

## Abstract

**Background:**

The increasing necessity for specialised training in primary care cancer research stems from GPs' pivotal role in cancer detection and holistic care coupled with the unique primary care context. This has led to the development of the PRiCAN Scholars Network, an initiative to enhance the research capabilities of Graduate Entry Medicine (GEM) students in RCSI University of Medicine and Health Sciences, Dublin, Ireland. This protocol outlines a proposal for the systematic development, implementation, and evaluation of a curriculum aimed at improving the primary care cancer research skills of this cohort.

**Methods:**

The curriculum development process will be guided by Kern’s six-step approach. Initial stages involve comprehensive needs assessments via surveys and focus groups to identify educational needs. Subsequently, targeted learning objectives and aligned educational strategies will be defined to maximise learning opportunities and impact. The curriculum’s impact will be evaluated in a pilot phase with selected students and faculty, utilising both qualitative and quantitative feedback to drive continuous improvements.

**Conclusion:**

This protocol describes a detailed method for establishing a primary care cancer research curriculum within the PRiCAN Scholars Network. Designed with a focus on sustainability and adaptability, the curriculum will be structured to develop and support a generation of medical professionals' literate in primary care research, contributing to the advancement of medical education and cancer research.

## Background

General practice is fundamental within the broader network of health care services known as “primary care”, which refers to all health care services located within the patient’s community ^1^. This includes General practitioners (GPs), GP nurses, Public Health Nurses (PHNs), community-based pharmacists, dental practitioners, physiotherapists, occupational therapists, psychotherapists, and medical social workers ^1^. In many healthcare systems across the world—such as those in Ireland, the UK, the Netherlands, Denmark, Australia, and New Zealand—primary care is the first point of contact with the health system.

GPs hold a unique position within the healthcare system due to the continuous nature of their relationship with patients, enabling them to observe subtle health changes over time. They act as the patient’s advocate, reviewing a high volume of patients with undifferentiated symptoms despite limited access to diagnostics and relying on their generalist medical knowledge to screen for significant pathology ^2^. GPs often tolerate a higher degree of uncertainty compared to their counterparts in secondary care.

Given this distinct role of primary care, and the unique characteristics of its population, and healthcare context, evidence supporting primary healthcare cannot be solely derived from secondary care research—i.e. research conducted by secondary care clinicians—primary care research is needed. This project aims to contribute to the development of primary care researchers equipped to answer the research questions posed by this multifaceted discipline, focusing on cancer due to its significant burden to public health.

### Role of primary care in cancer control

Cancer poses a significant public health challenge in Ireland, mirroring global trends in morbidity and mortality. In 2022, cancer was responsible for nearly 30% of all deaths, underlining the urgent need for comprehensive cancer control strategies ^3, 4^. The National Cancer Strategy 2017–2026 is Ireland’s response to this challenge, outlining objectives to improve prevention, early diagnosis, and patient care ^3, 5, 6^.

Primary care functions across the cancer care continuum, particularly in early detection and initial management which significantly impacts treatment success rates and patient survival ^7^. In Ireland, GPs bear the responsibility of detecting early signs and symptoms, carrying out diagnostic tests, and referring patients for specialist care ^8^. GPs can play a pivotal role in primary care research due to the quick turnover of patients, volume of patient contacts, management of multi-morbidity, and involvement in cancer screening programmes and health promotion campaigns.

Despite the crucial role of primary care in cancer control, there are areas requiring improvement, particularly in streamlining referral pathways, enhancing GP access to diagnostic tools, and refining integration of primary care into the broader cancer care network ^9^.

To address these challenges, primary care cancer research is needed, and as such, the capacity for primary care research generally must enhanced. Strengthening the role of primary care professionals in primary care research is central to this vision, requiring targeted investments in training, resources, and infrastructure to support GPs in their crucial role in cancer detection and management ^10^.

### Medical training in Ireland

Medical education in Ireland is structured across a continuum from undergraduate to postgraduate levels, emphasising medical sciences, clinical skills, and early patient engagement. Graduate Entry Medicine (GEM) programmes offer a condensed, four-year curriculum designed to leverage the students’ previous academic achievements ^11^. However, this intensive and clinically focused structure provides limited opportunities for in-depth research engagement ^11^. Existing self-directed courses and summer research projects, while beneficial, yield variable outcomes and do not consistently afford all students the depth of research experience required for contemporary medical practice or a future clinical academic career ^12^.

Enhanced student participation in research has been correlated with increased student knowledge and a sustained interest in research, highlighting the importance of such engagement ^13^. Longitudinal results of graduates from short-term research training programme found that 15% of students went on to conduct research as part of their professional responsibilities, suggesting the potential of such programmes to guide future physicians into research orientated careers ^14^.

Incorporating similar approaches within Ireland's medical training framework could profoundly enhance the quality and relevance of medical education, ensuring alignment with international standards and addressing the evolving needs of healthcare.

The long-established model of intercalated BSc degrees, allowing for focused study and research in specific medical fields, illustrates the benefits of integrating foundational research skills into medical training ^15^. Internationally, initiatives such as the Tisch Cancer Institute (TCI) Scholars Programme at the Icahn School of Medicine at Mount Sinai and the Scholars in Oncology-Associated Research (SOAR) programme at the University of Chicago demonstrate the value and impact of embedding research methodologies and projects within medical curricula ^12, 13^. The University of Edinburgh’s approach to integrating research skills development throughout the undergraduate medical curriculum sets a benchmark for research integration in medical education ^16^. These programmes enhance students' understanding of medical research, guide them in choosing future career specialties and research interests ^17^, and support research outputs ^18^.

### A specialised curriculum in primary care cancer research

Primary care based cancer research, critical for effective cancer control, has historically been underfunded compared to its laboratory and hospital-based counterparts, leading to significant gaps in knowledge and practice within the field ^19^. The Primary Care Research into Cancer (PRiCAN) research group aims to address these discrepancies by fostering collaboration among clinical academics, primary care researchers, clinicians, educators, and patient partners to develop a unified research agenda and optimise resource utilisation ^20^.

In response to these challenges and to align with the competencies for research prescribed by the Irish Medical Council ^21^, we propose the development of a specialised curriculum in primary care cancer research, embedded within the PRiCAN research group. This curriculum, which will aim to develop the primary care research capacity in health science students and primary care professionals, will initially be implemented in a group of GEM students—the “PRiCAN Scholars Network” (PSN).

The proposed curriculum for the PRiCAN Scholars Network will be designed to align with current educational standards ^22^, ensuring that the curriculum is both relevant for the evolving research demands of clinical practice and supported by best practices in medical education.

### Aim and objectives

The aim of this study is to develop a comprehensive research curriculum for the PRiCAN Scholars Network. We will achieve this aim via the following specific objectives:

1.   To conduct a needs assessment, systematically identifying the essential competencies and skills required in primary care cancer research.

2.   To develop an educational curriculum that incorporates the identified needs and utilises the most effective teaching strategies.

3.   To introduce the curriculum within the PRiCAN Scholars Network and evaluate its feasibility.

4.   To assess the impact of the curriculum on student research competencies and their engagement in primary care cancer research.

5.   To establish quality assurance processes to ensure the curriculum remains relevant, up-to-date, and continues to meet the educational standards and needs of primary care cancer research in Ireland.

## Methods

### Theoretical framework

This protocol for developing a curriculum in primary care cancer research for the PRiCAN Scholars Network is based on Kern’s six-step approach to curriculum development and is reported according to the “DoCTRINE Guidelines” for innovation in medical education ^23^. Kern’s model offers a structured, methodical framework that is well-suited for the development of a medical curriculum ^22^. Following Kern’s approach, we will begin with problem identification and general needs assessment, including a wide-ranging stakeholder engagement process crucial for understanding the specific educational requirements in primary care cancer research. The subsequent steps involve a targeted needs assessment of learners, setting specific goals and objectives, selecting appropriate educational strategies, implementing these strategies, and finally evaluating and refining the curriculum (see Table 1).

**Table 1.** Curriculum development process.

Step	Description
**Step 1**	General Needs Assessment: Surveys and interviews to understand current educational landscape in primary care cancer research.
**Step 2**	Targeted Needs Assessment: Student questionnaires and current curriculum review for in-depth understanding of specific learner needs.
**Step 3**	Goals and Objectives: Drafting and refining specific learning objectives for the curriculum.
**Step 4**	Educational Strategies: Development of curriculum framework and selection of effective teaching methods.
**Step 5**	Implementation: Pilot programme with small cohort before integration and coordination with existing curriculum.
**Step 6**	Evaluation and Feedback: Designing evaluation tools, conducting feedback sessions, and continuous curriculum refinement.
**Quality** **Assurance**	Review and Update: Regular curriculum review cycle and updates based on feedback, new research, and educational trends.

### Health education context

The curriculum development will take place within the Department of General Practice at RCSI University of Medicine and Health Sciences ^11^. RCSI is ranked as one of the top 250 universities worldwide and one of the top 50 for 'International Outlook' in the Times Higher Education World University Rankings 2023 ^23^. In 2023, RCSI retained 4,833 students with 330 graduating students ^23, 24^. The medical school offers both direct entry and graduate entry medicine programmes. The PSN will be established within the GEM programme, where students have previously completed at least a bachelor's degree, demonstrating foundational academic and literature skills, a level of commitment, problem-solving and communication skills.

The PSN is supported by RCSI’s Student Engagement and Partnership (StEP) programme which fosters collaboration between students, staff, and the RCSI community. The StEP programme was named as the winner of the 2022 ASPIRE-to-Excellence Award for Student Engagement ^25^.

### Curriculum development process

The research team will form a working group that will meet regularly to facilitate the educational research and implementation of the new curriculum. Representatives of the working group will include primary care and cancer researchers, medical students, PPI (public and patient involvement) representatives, and a medical education researcher. The curriculum development process will be completed over the six steps recommended by Kern *et al.* ^22^ (see Table 1), as outlined below.

### Step 1: “Problem identification and general needs assessment”

At the centre of this curriculum development initiative is the observation that primary care plays a key role in the diagnosis of cancer and the medical management of people living with cancer ^8^. This role is distinct from secondary care due to the low prevalence of undiagnosed cancer and the holistic focus of primary care. Therefore, this unique healthcare context demands tailored research to address cancer control in the community. However, the requisite primary care research will not materialise if future GPs and primary care professionals are taught medical research from a secondary care perspective, which focuses on managing specific diseases in small populations in high-resource, high-prevalence settings. Instead, a medical research curriculum that focus on primary care and population health study designs is needed.

Thus, the general needs assessment for a curriculum in primary care cancer research will include an analysis of the current approach and the ideal approach in medical education. The difference between the current and ideal approach represents the general needs assessment ^26^.

***Surveys.*** We will develop three distinct online surveys for medical students, faculty, and clinical professionals in Ireland to better understand how primary care cancer researchers are currently trained. The student survey will focus on educational experiences, areas of perceived under-preparation, and interest in cancer research topics. The faculty survey will explore teaching experiences, perceptions of student readiness, and areas needing curricular enhancement. The clinical professionals' survey will gather opinions on the readiness of graduates for primary care cancer research and suggestions for curriculum improvements. Each survey will be available open access via the Open Science Framework ^27^. They will consist of a mix of multiple-choice questions and open-ended question and will be distributed via institutional email lists, professional networks, and social media platforms. We aim to reach at least 100 medical students, 50 faculty members, and 50 clinical professionals with an anticipated response rate of approximate 40–50% in each subgroup, which is typical for higher educational surveys ^27^. The survey period will extend over four weeks, with bi-weekly reminders sent to encourage responses.

***Literature review.*** The ideal approach is focused to answer the question: “In an ideal world, how could primary care cancer researchers be trained?”. This will be determined by a review of the current literature on primary care cancer research. This review will help to identify both gaps and strengths in the current curricula in place internationally.

### Step 2: “Targeted Needs Assessment”

To better understand the needs related to approaches to primary care cancer research in medical curricula, specifically at RCSI for RCSI medical students, we will gather perspectives on the current state of primary care cancer research education from RCSI medical students, faculty, and clinical professionals. We will do this via a documentary analysis, interviews and focus groups.

***Documentary analysis.*** This involves collecting and analysing curriculum materials, syllabi, and teaching methodologies from different medical schools within Ireland and comparing them with international standards. The review will benchmark these curricula against international standards and recent advancements in primary care cancer research education. This will include a comparison with guidelines from recognised international medical education bodies and recent literature in the field of medical education. The review will be carried out by a team comprising medical educators, curriculum development experts, and representatives from the student body. This diverse team will ensure a comprehensive and balanced assessment of the current curricula.

***Interviews.*** We will conduct one-to-one interviews with the above-mentioned groups to gain insights into the specific needs and recommendations for the curriculum. We will target six key stakeholders, including RCSI senior medical educators, researchers in primary care cancer research, and experienced clinical professionals, ensuring a diverse representation of expertise and experience levels. Participation will be incentivised where appropriate to increase participation rates.

***Focus groups.*** We will also conduct focus groups with the above-mentioned groups to further understand the gaps that need to be addressed by the curriculum development process. We aim to recruit a total of 24 participants, divided evenly across at least 4 sessions ^28^. Each focus group session will last approximately 90 minutes, structured around predefined discussion points but also allowing for open-ended exploration of new ideas. The participants will include RCSI medical students, medical researchers and educators. The sessions will be facilitated by experienced moderators to ensure constructive discussions.

***Data analysis.*** Upon completion of data collection, we will analyse the data using various methods. Quantitative data from the surveys will be analysed using statistical software. We will use descriptive statistics to summarise responses and inferential statistics to identify significant trends and differences between groups. Transcripts from focus groups and interviews will be analysed using an open-source qualitative data analysis software such as Taguette ^29^. Thematic analysis will be employed to identify and categorize key themes, patterns, and insights relevant to curriculum development. We will synthesise findings from both the quantitative surveys and qualitative sessions to form a comprehensive understanding of the current landscape and needs in primary care cancer research education. This will inform the development of targeted educational strategies in subsequent curriculum development stages.

### Step 3: “Goals and objectives”

At this step, after general and targeted needs assessments have been completed, we will be able to define a list of broad and specific goals and objectives for the curriculum. These goals and objectives will reflect the gaps identified in the needs assessment and help to determine the content and learning methods of the curriculum. When defining the goals, we will use the “SMART” criteria, which ensures each goal is specific, measurable, achievable, realistic and within a certain period ^30^.

### Step 4: “Educational strategies”

After defining the curriculum goals, we will create a comprehensive curriculum framework, incorporating effective and innovative teaching methodologies tailored to the learning objectives and student needs. The curriculum framework will outline core content areas, instructional methods, and the integration of technology. This framework will be informed by the latest teaching methodologies and technological advancements in medical education. We will evaluate various teaching methods, such as interactive case studies, laboratory work, virtual simulations, and online learning platforms, to identify the most effective approaches for each curriculum component.

### Step 5: “Implementation”

After completing the above reviews and formulating a curriculum framework, we will implement the pilot curriculum within the PRiCAN Scholars Network, a small group of medical students (approximately 10–15 annually) who demonstrate exceptional interest and capability in primary care cancer research. These students will be selected through a rigorous application process that assesses motivation, academic excellence and research potential.

We will develop a strategy to integrate the curriculum into the existing medical studies of these students. This will include scheduling that accommodates the demands of their regular medical courses while allowing for meaningful engagement with the research curriculum. This may include flexible timetabling, use of online platforms for some components of the curriculum and providing support for time management. It will be necessary to allocate resources such as faculty mentors, research facilities, and learning materials specifically for this group.

To orient students, we will conduct an session to introduce the students to the curriculum objectives, expectations, and logistics. At this point we will begin the pilot phase. The pilot phase will be carefully monitored to assess the feasibility and impact of running the curriculum in parallel with standard medical studies. Adjustments will be made as needed based on initial observations and feedback.

### Step 6: “Evaluation and feedback”

Various evaluation and feedback mechanisms will be established to comprehensively evaluate the impact of the curriculum within the PRiCAN Scholars Network and develop a robust feedback system to continuously refine the programme. These evaluation tools will be tailored to the small cohort size and the specialised nature of the curriculum. This may include pre- and post-curriculum assessments to gauge knowledge and skills acquisition, regular reflective journals or portfolios to track progress, and specific project evaluations to assess research competencies. We will implement surveys and feedback forms that are designed to capture detailed qualitative and quantitative feedback from both students and faculty. Utilize online platforms for continuous and convenient feedback collection. This can include discussion forums or digital suggestion boxes where students can share thoughts and feedback asynchronously. We will also schedule regular feedback sessions, including one-on-one meetings and small group discussions, to gather in-depth feedback from the students. These sessions will allow for a more nuanced understanding of the students' experiences and challenges.

We will use the insights gained from these evaluation and feedback mechanisms to make ongoing adjustments and improvements to the curriculum. Given the dynamic nature of the field and the high calibre of the students, the curriculum should be agile and responsive to both emerging trends in cancer research and specific learning needs of the cohort.

Longer term, we aim to implement a system to evaluate the curriculum’s long-term impact on students’ career trajectories and research contributions, providing valuable insights for further programme development ^31^. To do this, we will apply the RE-AIM framework, used to plan and implement public health and behaviour change innovations, encompassing five stages – Reach, Effectiveness, Adoption, Implementation, and Maintenance/Sustainment – to adopt and implement sustainable interventions ^32^.

### Quality assurance and enhancement

To ensure the curriculum is consistently up to medical school standards, there will be a structured review cycle that is tailored to the unique nature of the programme. This cycle should involve semi-annual reviews to align with the academic year. An independent committee comprising medical education experts, primary care cancer research specialists, and external academic advisors will form the review committee. We would also like to include alumni of the programme and representatives from relevant healthcare sectors, in the review process to provide diverse perspectives and enhance the curriculum's relevance to real-world practices

This committee will conduct periodic assessments of the curriculum to ensure it maintains high standards and remains at the forefront of educational excellence.

### Curriculum update procedures

We will develop a protocol for regularly updating the curriculum based on direct feedback from students and faculty, insights from the review committee, and evaluations. This process should be agile to allow for timely implementation of changes.

Simultaneously, we will implement a mechanism for continuously monitoring and integrating new research findings, technological advancements, and educational trends into the curriculum. This may involve a dedicated team or individual responsible for staying up to date with the latest developments in primary care cancer research and medical education.

Additionally, we would implement a quality improvement process, where data from regular evaluations and feedback are used not only to update the curriculum but also to refine teaching methodologies, assessment strategies, and overall programme structure.

Comprehensive documentation of all changes and updates to the curriculum will be maintained, including the rationale behind these changes. This practice will ensure transparency and provide valuable insights for future curriculum development.

## Conclusion

The development of this specialised curriculum in primary care cancer research is anticipated to yield several key outputs. Firstly, it will create an educational framework that fills the current gap in primary care cancer research training in Ireland, particularly tailored for a cohort of Graduate Entry Medicine students through the PSN. This framework will include a comprehensive set of learning outcomes, educational resources and assessment tools. Additionally, the curriculum is expected to foster a stronger foundation in research among students, preparing them for future roles as innovators and leaders in healthcare.

### Risks and opportunities

The curriculum’s impact will be largely dependent on the engagement and feedback from participants, which could vary. We plan to target a large cohort of individuals during the data collection phase to ensure substantial feedback is obtained. Furthermore, the specific focus on primary care cancer research within the GEM cohort at RCSI may limit the transferability of the curriculum to other settings or cohorts; however the detailed and transparent approach to its design, implementation and evaluation may service as an easily adaptable model for research development in other contexts Additional challenges include research planning complexities, lack of infrastructure, difficulties in engaging healthcare professionals, and barriers to knowledge translation ^33^.

### Implications for practice, policy, education, and research

The development and implementation of this curriculum has the potential for broad implications. In practice, it will provide a defined cohort of GEM students with specialised research skills, enhancing the capabilities of future primary care practitioners in cancer research. In terms of policy and education, this initiative can serve as a model for curriculum development in other specialised areas of healthcare, promoting a more research-intensive approach in medical training ^34^. The programme also aims to build a community of academic primary care professionals, encouraging further studies into effective educational strategies in medical education. Ultimately, the programme can act as a catalyst for building a research-oriented community in primary care ^35^.

### Dissemination

The dissemination of the new primary care cancer research curriculum developed through the PRiCAN Scholars Network will be multi-layered and targeted to reach a wide array of stakeholders in both the academic and healthcare sectors.

Upon project completion, findings and curriculum details will be published in high-impact medical education and primary care journals. The project will be presented at both national and international medical education conferences to reach educators and policymakers, ensuring the curriculum's innovations and effectiveness are widely recognised.

We will engage with educational policymakers and accrediting bodies to advocate for the integration of key components of the curriculum into broader medical training programmes. We will host workshops and webinars for medical educators and administrators from other institutions. These sessions can serve as a guide for adopting similar curricular structures in their programmes, fostering a community of practice around innovative medical education. We will also engage with graduates and current students as ambassadors to share their experiences and insights from the curriculum with their professional networks, thus creating a ripple effect in the medical community. These initiatives could potentially influence national and international medical education standards.

We will also partner with healthcare organisations and primary care networks to demonstrate how the curriculum aligns with current clinical practices and enhances the research capacity in primary care settings. This can facilitate the translation of educational outcomes into practical healthcare improvements.

To reach a broader audience, we will utilise digital platforms, including dedicated websites and social media channels, to share curriculum resources, such as syllabi, teaching materials, and assessment tools. This open-access approach can facilitate adaptation and adoption by other medical schools and training programmes.

By implementing this comprehensive dissemination strategy, the new curriculum's impact can be extended significantly, influencing medical education practices, enhancing primary care research capacity, and ultimately contributing to improved healthcare outcomes.

### Conclusion

This protocol presents a strategic approach to developing a curriculum that addresses a significant need in medical education – the integration of research training in primary care cancer research. By doing so, it aims to not only enhance the skills and competencies of medical students in this critical area but also to provide a template for similar initiatives in other medical disciplines. The expected outputs, despite potential limitations, have the capacity to influence practice, policy, education, and research significantly. The dissemination of the outcomes will further contribute to the ongoing evolution of medical education, aligning it more closely with the demands and challenges of contemporary healthcare.

## Ethical approval and written consent

This research will be conducted in full compliance with the ethical guidelines and policies of the Royal College of Surgeons in Ireland (RCSI) and reviewed by the Research Ethics Committee. A plan for informed consent and ethics specific to each proposed work package (mapped to Kern’s Six Steps) is here outlined:

- **Step 1 (Surveys)**: Following peer review of the protocol, research ethics committee approval will be sought from the RCSI Research Ethics Committee to conduct the surveys. Informed, written consent will be obtained electronically as part of the step in any non-anonymous online surveys. Sample consent forms are available via the Open Science Framework.
- **Step 1 (Literature review)**: No research ethics committee approval is required for a literature review.
- **Step 2 (Documentary analysis)**: Research ethics committee approval is not required for a documentary analysis.
- **Step 2 (Interviews)**: Following completion of Step 1, research ethics committee approval will be sought from the RCSI Research Ethics Committee to conduct both the interviews and the focus groups. Informed written consent will be obtained in advance of the interview commencement. Sample consent forms are available via the Open Science Framework.
- **Step 2 (Focus groups)**: Following completion of Step 1, research ethics committee approval will be sought from the RCSI Research Ethics Committee to conduct both the interviews and the focus groups. Informed written consent will be obtained in advance of conducting the focus groups. Sample consent forms are available via the Open Science Framework.
- **Step 3 (Goals and Objectives):** Research ethics committee approval is not required for a planning and design process, as this is not a research study, nor does it involve human subjects or data subjects.
- **Step 4 (Educational Strategies):** Research ethics committee approval is not required for a planning and design process, as this is not a research study, nor does it involve human subjects or data subjects.
- **Step 5 (Implementation):** Research ethics committee approval is not required for the implementation of a medical education curriculum, as this does not constitute a research study.
- **Step 6 (Evaluation and Feedback):** Research ethics committee approval is not required for the evaluation and feedback step, as this does not constitute a research study, but rather a routine curriculum evaluation process adhering to the relevant policies within RCSI University of Medicine and Health Sciences.

## Patient and Public Involvement

Ms. Carmel Geoghegan, a patient and public involvement (PPI) contributor for the Primary Care Research into Cancer (PRiCAN) research group, offered feedback on this protocol. PPI contribution on this project—in the conduct, analysis and dissemination of the research—will continue via the SPARC (Stakeholder Group for Primary Care) PPI panel.

## Data Availability

This article is a research protocol, and as such, does not include any associated datasets. Datasets generated during the research will be made permanently available in an open-access repository, the Open Science Framework, ensuring they are accessible to the public and other researchers. The following extended data (i.e., supplementary files) are available via the Open Science Framework at the following: **PRiCAN Development Programme: PSN Protocol,** https://doi.org/10.17605/OSF.IO/HGDYU ^27^. < PSN_FocusGroup_Consent_v01> *Consent form for Focus Group Participation* < PSN_FocusGroup_PreParticipation Info _v01> *Pre-participation information for Focus Group*s < PSN_FocusGroup_DiscussionGuide_v01> *Discussion guide for Focus Groups* < PSN_Interview_Consent_v01> *Consent form for Interview* < PSN_Interview_Guide_v01> *Guide to Interview* < PSN_Interview_PreParticipationInfo_v01> *Pre-participation information for interview* < PSN_Survey_ClinicalProfessionals_v01> *Survey for Clinical Professionals* < PSN_Survey_Faculty_v01> *Survey for Medical Faculty* < PSN_Survey_Student_v01> *Survey for Medical Students* Copyright: © 2024 Verlaque L *et al.* This is an open access article distributed under the terms of the Creative Commons Attribution License (CC-By Attribution 4.0 International), which permits unrestricted use, distribution, and reproduction in any medium, provided the original work is properly cited.
