# Supplementary material for: Protocol to develop a specialised curriculum in primary care cancer research in an Irish medical school
Source: HRB Open Res. 2024 Oct 8;7:64. [Version 1] doi: 10.12688/hrbopenres.13911.1 (PMC11803193; doi:10.12688/hrbopenres.13911.1)
Supplement: Peer review history

# Reviewer report: Reviewer response for version 1

**DOI:** 10.21956/hrbopenres.15259.r42918

**Published:** 2024-12-16

**Authors:** Dorothy Lombe

**Affiliations:** 1 Department of Radiation Oncology, MidCentral District Health Board, Palmerston, New Zealand

Title and Abstract: The title and abstract are representative of the content of this manuscript. They summarise the intended content well.

 References: The references utilised are appropriate and within reasonable time period

 Background: the background of the problem is well articulated and provides sufficient precedence for this study. The authors rightfully correlated the role of primary care in cancer control. Their observations are accurate.

 Methods: The methods being proposed are appropriate. in table 1, the authors may consider elaborating step 2 and the role of faculty in this step.

 General comments: This is a well designed study protocol and i wish the authors success. I look forward to the results of its conduct.

Is the study design appropriate for the research question?

Yes

Is the rationale for, and objectives of, the study clearly described?

Yes

Are sufficient details of the methods provided to allow replication by others?

Yes

Are the datasets clearly presented in a useable and accessible format?

Not applicable

Reviewer Expertise:

Cancer

I confirm that I have read this submission and believe that I have an appropriate level of expertise to confirm that it is of an acceptable scientific standard.

# Reviewer report: Reviewer response for version 1

**DOI:** 10.21956/hrbopenres.15259.r42913

**Published:** 2024-11-08

**Authors:** Kim Benstead

**Affiliations:** 1 Gloucestershire Hospitals, NHS Foundation Trust, Cheltenham, UK

This paper describes a protocol to develop, implement and evaluate a curriculum aimed at enhancing the research capabilities of Graduate Entry Medicine students in a single institution using the Kern's six-step approach. The paper is well written. The methodology for all elements, the development, implementation and evaluation are appropriate and thorough. The presence of public and patient involvement representatives on the working group is very positive. Mechanisms for future updating of the curriculum are well described. The strategy for dissemination of the curriculum is comprehensive.

 The choice of the pilot group from the Graduate Entry Medical Programme rather than the undergraduate stream could be discussed more thoroughly. The authors discuss the benefits of intercalated BSc degrees. The participants in the Graduate Entry Programme will have already have undertaken a degree likely to be a BSc and may have already undertaken training in research and small research projects as part of those degrees. The benefit seen in the undergraduate stream might therefore be expected to be greater than the graduate stream.

 What proportion of the students choose a career in primary care from the graduate stream compared with the undergraduate stream and do the authors think this might be relevant? I note that the survey for medical students is aimed at both the undergraduate and graduate streams. It might be expected that this will result in the curriculum being applicable to both but not tailored to the pilot study group who are on the graduate entry scheme. Will the results for these two groups be analysed separately?

Is the study design appropriate for the research question?

Yes

Is the rationale for, and objectives of, the study clearly described?

Yes

Are sufficient details of the methods provided to allow replication by others?

Yes

Are the datasets clearly presented in a useable and accessible format?

Not applicable

Reviewer Expertise:

Curriculum design

I confirm that I have read this submission and believe that I have an appropriate level of expertise to confirm that it is of an acceptable scientific standard.
